# Artificial Auricular Cartilage Using Silk Fibroin and Polyvinyl Alcohol Hydrogel

**DOI:** 10.3390/ijms18081707

**Published:** 2017-08-04

**Authors:** Jung Min Lee, Md. Tipu Sultan, Soon Hee Kim, Vijay Kumar, Yeung Kyu Yeon, Ok Joo Lee, Chan Hum Park

**Affiliations:** 1 Nano-Bio Regenerative Medical Institute, College of Medicine, Hallym University, Chuncheon 200-702, Korea; yiyi1124@gmail.com (J.M.L.); tipubge@yahoo.com (M.T.S.); soonheekim@gmail.com (S.H.K.); vijay10187@gmail.com (V.K.); demaniaba@gmail.com (Y.K.Y.); vudckd@hanmail.net (O.J.L.); 2 Department of Otorhinolaryngology-Head and Neck Surgery, Chuncheon Sacred Heart Hospital, Hallym University College of Medicine, Chuncheon 200-704, Korea

**Keywords:** silk fibroin, 3D printer, auricular cartilage

## Abstract

Several methods for auricular cartilage engineering use tissue engineering techniques. However, an ideal method for engineering auricular cartilage has not been reported. To address this issue, we developed a strategy to engineer auricular cartilage using silk fibroin (SF) and polyvinyl alcohol (PVA) hydrogel. We constructed different hydrogels with various ratios of SF and PVA by using salt leaching, silicone mold casting, and freeze-thawing methods. We characterized each of the hydrogels in terms of the swelling ratio, tensile strength, pore size, thermal properties, morphologies, and chemical properties. Based on the cell viability results, we found a blended hydrogel composed of 50% PVA and 50% SF (P50/S50) to be the best hydrogel among the fabricated hydrogels. An intact 3D ear-shaped auricular cartilage formed six weeks after the subcutaneous implantation of a chondrocyte-seeded 3D ear-shaped P50/S50 hydrogel in rats. We observed mature cartilage with a typical lacunar structure both in vitro and in vivo via histological analysis. This study may have potential applications in auricular tissue engineering with a human ear-shaped hydrogel.

## 1. Introduction

Auricular cartilage is elastic cartilage with a collagen network of type-II collagen and highly sulfated glycosaminoglycan (sGAG) [1]. Although auricular cartilage has excellent mechanical strength, once injured, its lack of intrinsic self-repair and regenerative abilities make self-healing difficult [2]. Different types of auricular deformities due to congenital anomalies, trauma or burns remain challenging to address [3]. Tissue engineering and reconstruction has been considered the most promising approach to address these types of abnormalities. Chondrogenic differentiation following treatment with appropriate biochemical factors is a prerequisite for successful cartilage tissue engineering. A three-dimensional (3-D) porous scaffold is also required to provide a suitable environment for chondrogenesis and new cartilage-specific extracellular matrix (ECM) formation [4]. Furthermore, several studies demonstrated that lack of control over engineered tissue leads to the failure of cartilage tissue engineering. Therefore, advanced studies have focused on the progress of 3D printing-based tissue engineering to produce the precise structure of the human tissue. The use of computer-aided design/manufacturing (CAD/CAM) is a conventional method to control 3D architecture of the scaffold [5,6,7,8,9,10].

The selection of scaffolds is very important for tissue replacement therapies, including cartilage regeneration [11]. The ideal biomaterials for 3D ear-shaped cartilage should have sufficient elasticity, flexibility, mechanical strength, and physical stability to maintain their shape [10]. Besides these characters, biocompatibility, bioresorbability, and porosity are also required for scaffolds to encourage cell attachment, interior chondrogenesis, and neo-tissue regeneration [6]. Several types of scaffolds of different polymers have been reported for use in cartilage tissue engineering, including gelatin, polycaprolactone (PCL), alginate, collagen I, polylactic acid (PLA), bacterial nanocellulose (BNC), and polyglycolic acid (PGA) [5,6,7,8,9,10]. However, cartilage tissue engineering using a scaffold is frequently associated with some complications. For example, high concentrations of PLA reduce cell proliferation, while acellular cartilage sheets (ACSs) are associated with concerns regarding availability and a non-porous structure [9,10]. Therefore, to overcome the currently existing problems with cartilage tissue engineering, we evaluated an ear-shaped hydrogel composed of SF and PVA for use in auricular cartilage tissue engineering.

Hydrogels are hydrophilic 3D polymeric network with high swelling ratios in water. Due to their high moisture content and elasticity, they have been shown to better ability to mimic human tissue than other synthetic biomaterials [12]. Hydrogels have been extensively used in a vast range of biomedical applications, mainly in drug delivery and tissue engineering [13,14,15,16,17]. Some of the biological and mechanical properties of hydrogels are comparable with those of auricular cartilage such as cellular immobilization, a reasonable flexibility, and an efficient porous structure [18,19].

SF, a natural protein polymer, has been widely used in a number of biological and biomedical fields owing to its biodegradable, biocompatible, non-toxic, and non-immunogenic properties [20,21,22,23]. It has been used as a biomaterial in various forms, such as fibers, films, hydrogels, and 3D scaffolds [24,25,26]. Among theese forms, recently, silk hydrogels have been gained significant attention in the field of tissue engineering, as these hydrogels provide a large surface area for cell growth, proliferation, adhesion, and migration [27,28,29,30,31]. Several recent studies have been conducted on silk hydrogel for its application as a cell cuture matrix, a drug delivery system, a gene delivery system, and an artificial skin [32,33,34]. In addition, numerous studies have shown that SF is a promising biomaterial for use in cartilage tissue engineering [4,35,36].

PVA is a synthetic hydrophilic, semi-crystalline, and non-toxic polymer [37]. It is widely used in cartilage tissue engineering because it has a mechanical strength almost similar to that of native cartilage [38]. PVA-based hydrogels are developed via different crosslinking process such as physical, chemical, and irradiation crosslinking. Physical crosslinked hydrogels (freeze-thawing hydrogels without toxic residues) offer improved mechanical properties compared to hydrogels made by chemical (have toxic residues) and irradiative (expensive) cross linking techniques [39]. Owing to its biocompatibility, ease of processing, and suitable mechanical strength, freeze-thawing PVA hydrogels have been extensively used in many tissue engineering applications, particularly in articular cartilage tissue engineering [40,41,42,43]. In addition, the viscoelastic properties of physically cross-linked freeze-thawed PVA hydrogels are comparable with those of articular and meniscal cartilage, making them remarkably attractive biomaterials for tissue engineering applications [44,45]. Because freeze thawing PVA is inexpensive and non toxic, we selected it as a suitable material for this study.

In the present study, we engineered an intact auricular cartilage using an SF and PVA composite ear-shaped hydrogel in vivo. Several types of hydrogels were fabricated with different ratios of SF and PVA. Swelling ratio, tensile strength, pore size, thermogravimetric analysis (TGA), scanning electron microscope (SEM), fourier transform infrared spectroscpy (FTIR), and cell viability (CCK-8) analyses were also performed to evaluate the physical, mechanical, and biological properties of each hydrogel. We also assessed in vitro and in vivo chondrogenesis using histological evaluation techniques.

## 2. Results

### 2.1. Structural and Morphological Characteristics

**Figure 1:**
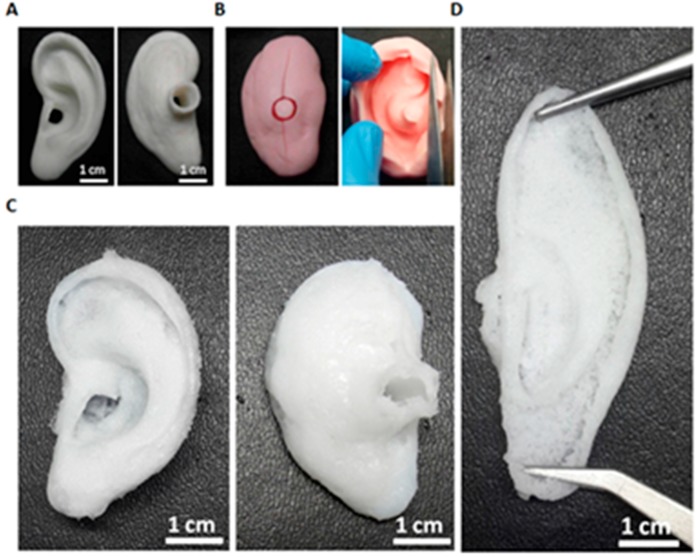
Gross view of the polyvinyl alcohol/ silk fibroin (PVA/SF) ear-shaped hydrogel. (**A**) polylactic acid (PLA) molds (front and back views); (**B**) Silicone molds (closed and open views); (**C**) PLA/SF ear-shaped hydrogel (front and back views) (**D**) PLA/SF ear-shaped hydrogel with physical stretching.

Figure 1 shows the microscopic appearances of the PLA mold, silicone mold, and the PVA/SF ear-shaped hydrogel. The PVA/SF ear-shaped hydrogel shared morphological and physical properties with the natural human ear, such as structure, flexibility, elasticity, and softness (Figure 1). In tissue engineering, controlling the shape is a major challenge, particularly for tissues with fragile 3D structures [46]. In this study, we prepared a PLA positive mold using a 3D printer following the methods in previous studies to produce a precise 3D ear-shaped hydrogel [2,6,7,10]. A corresponding silicone negative mold was also fabricated to construct the desired PVA/SF hydrogel. PVA/SF-derived ear-shaped hydrogel had a 3D structure similar to that of the positive mold even after exposing it to physical stretching (Figure 1D).

**Figure 2:**
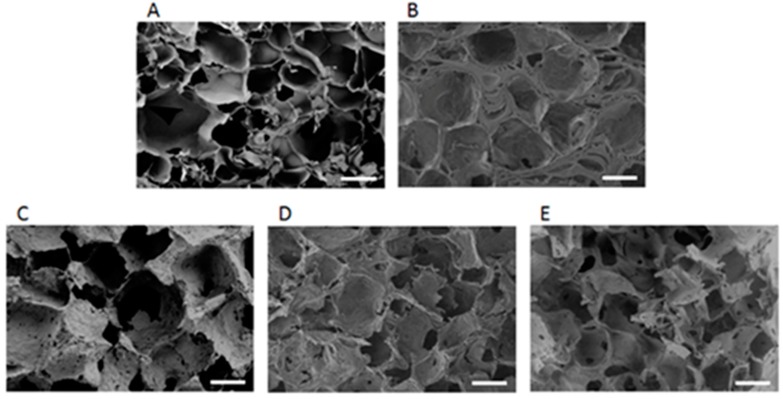
The cross-section scanning electron microscope (SEM) images of hydrogels. (**A**) S100; (**B**) P100; (**C**) P25/S75; (**D**) P50/S50; (**E**) P75/S25. Scale bars = 100 μm.

To optimize a suitable PVA/SF composite hydrogel, we fabricated various types of hydrogels using different combinations of PVA and SF. The cross-sectional SEM images of the five types of hydrogels are shown in Figure 2. Each hydrogel had a different pore size and pore structure. The S100 hydrogel contained interconnected irregular pores with a thin wall between pores (Figure 2A), whereas PVA100 contained closed pores with a thick wall between pores (Figure 2B). The P50/S50 hydrogel had a uniform appearance and sponge-like structures on the surface unlike the two other (P25/S75 and P75/S25) composite hydrogels (Figure 2C–E). These observations indicated that the P50/S50 hydrogel was highly interconnected between the pores.

### 2.2. Mechanical Properties, Swelling Ratio, and Pore Size

**Figure 3:**
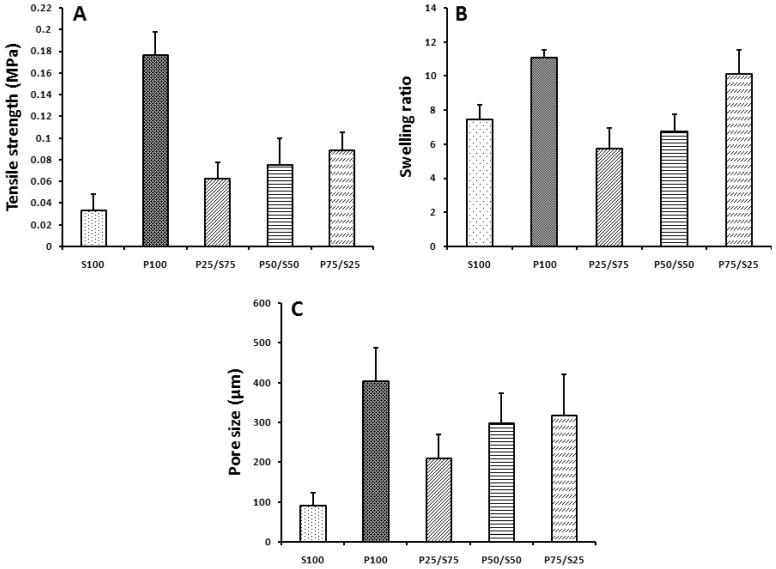
(**A**) Tensile strength, (**B**) swelling ratio, and (**C**) pore size of the hydrogels.

Figure 3 represents the mechanical properties of the hydrogels. The P100 hydrogel had the highest tensile strength of 0.1765 ± 0.0208 MPa whereas the S100 hydrogel had the lowest tensile strength, 0.0326 ± 0.0149 MPa. The blended P50/S50 hydrogel had an intermediate tensile strength of 0.0751 ± 0.0246 MPa. The tensile strength of the SF hydrogels increased gradually as the PVA concentration increased (Figure 3A).

As shown in Figure 3B, the homogeneous hydrogels of SF and PVA had higher swelling ratios than the composite hydrogels. Among the fabricated hybrid hydrogels, the P25/S75 and P75/S25 hydrogel had the lowest and highest swelling ratios, 5.48 ± 0.48 and 1.91 ± 0.58 respectively, whereas the swelling ratio of the P50/S50 hydrogel was intermediate at 3.19 ± 0.79. The pore size of the fabricated hydrogel is related to the concentration of the PVA in the hydrogels. As the PVA concentration increased, the pore size of the composite hydrogel increased (Figure 3C).

### 2.3. FTIR and TGA

**Figure 4:**
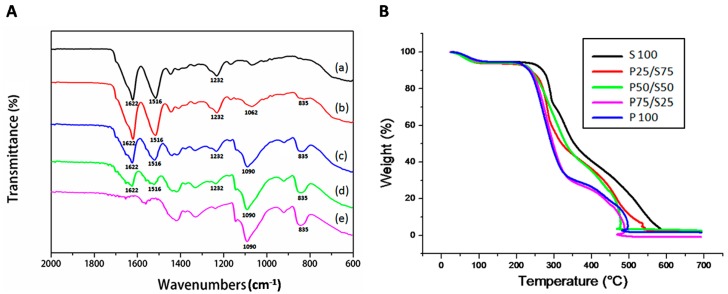
(**A**) FTIR spectra; (**a**) S100; (**b**) P25/S75; (**c**) P50/S50; (**d**) P75/S25; (**e**) P100; and (**B**) Thermogravimetric analysis.

The FTIR peak spectra results for the PVA/SF composite hydrogels are shown in Figure 4A. In this experiment, SF peaks were observed at 1622, 1516, and 1232 cm^−1^, representing amide І, amide II, and amide III, respectively. These peaks were also found in the PVA/SF composite hydrogels but were absent in the PVA hydrogel. The PVA spectrum contained peaks at 1090 cm^−1^ (C–O, out-of-plane bonding), and 835 cm^−1^ (C–C bonding), but in the P75/S25 hydrogel, the PVA (C–O bonds) peak was shifted to 1068 cm^−1^. The peak height and area were directly proportional to PVA/SF ratio.

The thermal stability of the different PVA/SF hydrogels was analyzed, and the results are shown in Figure 4B. The hydrogel of 100% SF (S100) exhibited the onset of degradation at 250 °C, whereas the 100% of PVA (P100) hydrogel exhibited early onset degradation at 200 °C. The P50/S50 hydrogel showed an intermediate onset of degradation at 220 °C. These results indicated that the addition of SF to PVA increased the thermal stability of the blended hydrogel.

### 2.4. Biocompatibility of the Hydrogels

**Figure 5:**
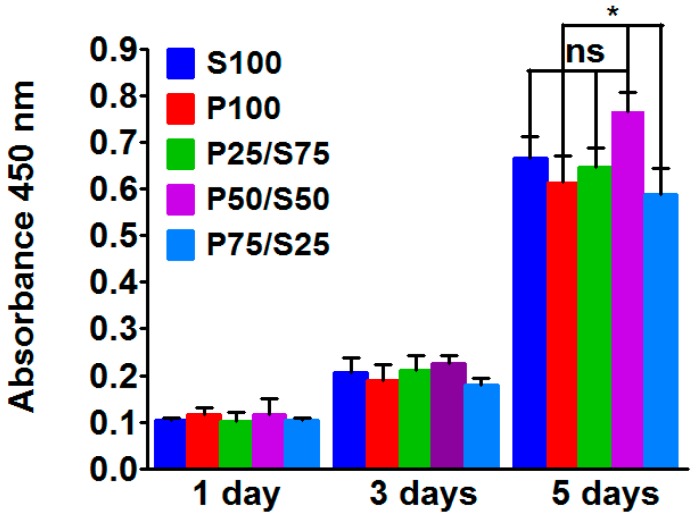
CCK-8 assay after culturing rat ear chondrocytes on hydrogels for 1, 3 and 5 days. Significant differences are presented as * *p* < 0.05, and ns indicates not significant.

To evaluate the biocompatibility of the constructed hydrogels, isolated chondrocytes were culture on each hydrogel, and cell viability was measured using the CCK-8 assay. The graph of the CCK-8 results presents the absorbance at 450 nm after culturing for one, three and five days. The metabolic activity of chondrocytes on the different types of hydrogels gradually increased from day one to five (Figure 5).

The P50/S50 hydrogel showed significantly more cell growth compared to the P100 and P75/S25 hydrogel after five days of culture. These results indicated that the P50/S50 hydrogel provided a better environment for chondrocyte growth. However, cells on P75/S25 showed the lowest proliferation among the composite groups after five days of culture.

### 2.5. In Vitro Chondrogenesis

**Figure 6:**
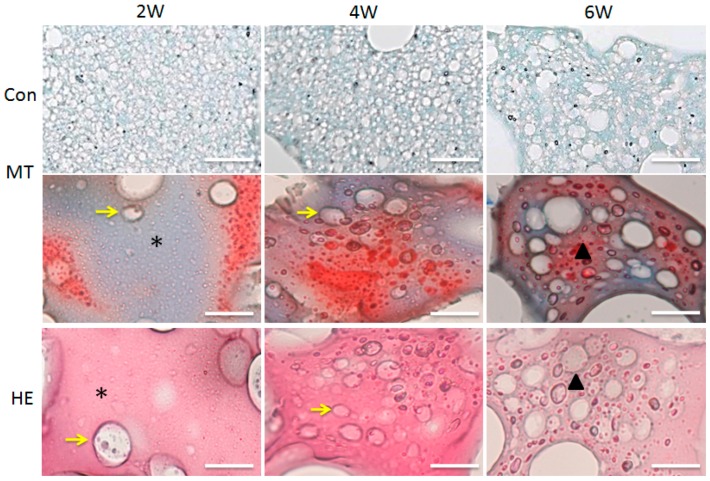
Histological examination of in vitro chondrogenesis: At 2 weeks, the cartilaginous matrix (black stars), immature lacuna (yellow arrows). At 4 weeks, the cartilaginous matrix contained a larger number of lacunae (yellow arrows). At 6 weeks, auricular cartilage (triangles) was visible. Scale bars = 200 μm.

Masson’s Trichrome (MT) and Hematoxylin and eosin (H&E) staining were performed after two, four, and five weeks of chondrocytes culture on the P50/S50 hydrogel. Both MT and H&E staining results showed that the lacunar structure and neo-cartilage formation appeared in the two-week-old specimens (Figure 6). The number of lacuna were increased from four weeks to six weeks (Figure 6), which means that cartilage maturity gradually increased over time. A higher amount of residual hydrogel components was observed after two weeks of culture, whereas a fewer amount was found after six weeks of culture.

### 2.6. In Vivo Engineered Cartilage

**Figure 7:**
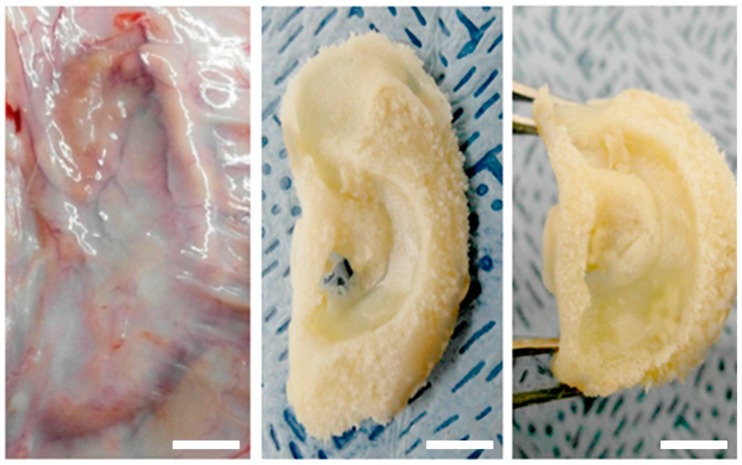
Engineered ear-shaped cartilage: Surgical view of 6 weeks after implanting, after harvesting, and after subjecting to physical stress. Scale bars = 1 cm.

**Figure 8:**
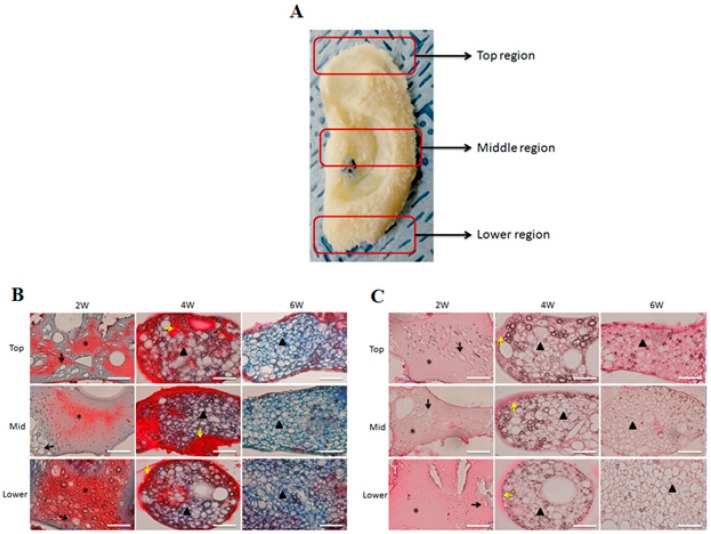
Histological examination of engineered ear-shaped cartilage: (**A**) Regions for the histological evaluation of engineered cartilage: the top, middle, and lower regions were evaluated for cartilage formation. (**B**) Masson’s Trichrome (MT) staining: (**C**) Hematoxylin and eosin (H&E) staining. At 2 weeks, there was a various-shaped immature lacunar structure (black arrows) within the cartilaginous matrix (stars). At 4 weeks, there was an increase in the number of lacunar structures surrounded by cytoplasmic constituents (yellow arrows) and immature cartilage (triangle). At 6 weeks, mature cartilage (triangle) with a typical chondrocyte lacunar network was visible. Scale bars = 200 μm.

To reduce the chances of immunogenic rejection, isolated chondrocytes from rats were seeded on the hydrogel and then implanted into the same rat. The ear-shaped engineered cartilages were harvested after six weeks of culture and were found to be phenotypically and mechanically stable (Figure 7). These results indicated that the PVA/SF composite ear-shaped hydrogel had sufficient mechanical strength to maintain its original shape throughout the culture period. We hypothesized that there would be no graft rejection or immunogenic reactions with the harvested ear-shaped engineered cartilages. The histological findings indicated that there was no inflammatory response, including neutrophil or lymphocyte aggregation, in the in vivo engineered cartilage samples (Figure 8). This result indicated that the PVA50/S50 hydrogel did not elicit any immunogenic response during the experimental period.

The histological evaluation of the engineered cartilage was carried out on three different parts of the implants, as shown in Figure 8A. MT staining six weeks after implanting revealed a much stronger blue-stained collagen deposition compared to that in the four-week-old samples. This observation indicated that there was more cartilage formation in the six-week cultured ear-shaped hydrogel. The cultured implants consistently showed neo-cartilage formation on the PVA/SF ear-shaped hydrogel (Figure 8B,C). The developed cartilaginous matrix showed a significant number of chondrocytes with partially mature lacuna at two weeks after implanting. In the 4-week implants, a typical chondrocyte lacunar structure was formed that was surrounded by cytoplasmic constituents. However, the development of cartilage, deposition of ECM, and the number of lacuna continuously increased during the culture period. We also observed higher amounts of ECM formation and proper organized elastic fibers after six weeks of culture. The cartilaginous matrix at 6 weeks was completely converted into mature auricular cartilage with the typical lacunar network. Histological analyses of the implants also revealed that the number of chondrocytes per unit area significantly decreased with the maturation of cartilage from two to six weeks.

## 3. Discussion

The current study demonstrates the in vitro and in vivo auricular cartilage regeneration using PVA/SF composite ear-shaped hydrogel. Previously, it was reported that silk-derived hydrogel is an efficient bio-material for chondrogenesis and ECM deposition, including cartilage-specific sGAG [47]. Likewise, the mechanical strength of the PVA-derived hydrogel is reasonably supportive for cartilage tissue engineering [40]. However, to the best of our knowledge, no study has used silk and PVA-blended hydrogels for auricular cartilage tissue engineering. Therefore, we designed this study to develop a better strategy for auricular cartilage tissue engineering using silk fibroin and PVA hydrogel. We observed that the PVA/SF ear-shaped hydrogel exhibited a structural resemblance to the natural human ear (Figure 1). After being subjected to physical stretching, the PVA/SF-derived hydrogel also retained a similar 3D structure with the positive mold (Figure 1D). Thus, the PVA/SF hydrogels developed in our study could be used to overcome the problem associated with maintaining a 3D structure for both in vitro and in vivo chondrogenesis [46]. Previously, it was reported that an inappropriate porous structure of a scaffold limits cell proliferation and chondrogenesis [48]. In the present study, we observed that the P50/S50 porous hydrogel was uniform in appearance and had sponge-like structures on its surface (Figure 2D). The introduction of SF to PVA resulted in more interconnected open pores. Pore size plays a vital role in tissue engineering with hydrogels regarding the regulation of cell behavior and infiltration into the hydrogel [49,50]. Cell infiltration depends on different parameters, including pore size, pore interconnectivity, surface morphology, and oxygen diffusion. A maximum cell infiltration into 150–200 µm porous scaffolds has been reported [51]. In this study, the porosity of the hybrid hydrogels increased as the PVA concentration increased (Figure 3C). Therefore, the engineering approach developed in the current study avoids the need for secondary treatment (such as laser or microwave treatment) to increase the porosity [9].

In the present study, we determined the tensile strength of the hydrogels. It was observed that the tensile sternth of the SF hydrogels increased gradually as the PVA concentration increased (Figure 3A). Water uptake and the swelling ratio are important properties of a biomaterial for use in tissue engineering. Water uptake and the swelling ratio are alos directly related to a material’s ability to provide an environment for cell growth and its mechanical properties and the tensile strength [52]. The swelling ability of hydrogel plays a critical role in terms of regulating the diffusion rate of nutrients and exchange of wastes within the hydrogel [53]. Although higher swelling ratios are favorable for cartilage matrix production, they can also reduce the mechanical properties of the hydrogels [54]. We performed a swelling ratio analysis of all our fabricated hydrogels. It was found that the homogeneous hydrogels of SF and PVA had higher swelling ratios than the composite hydrogels (Figure 3B), possibly because of the hydrophylic nature of the PVA or the increased posrosity within the composite hydrogel structure. In order to evaluate the chemical structure of the hydrogels, we carried out an FTIR analysis. SF exhibited peaks at 1622, 1516, and 1232 cm^−1^ on its FT-IR spectra. These peaks were also found in the PVA/SF composite hydrogels but were absent in the PVA hydrogel (Figure 4A). These FTIR results were similar to our previously reported result [55]. We also measured the thermal stability of the different PVA/SF hydrogels and found that the addition of SF to PVA increased the thermal stability of the blended hydrogel (Figure 4B).

In this study, we also evaluated the effect of the constructed hydrogels on the cell growth of isolated chondrocytes using the CCK-8 assay. We found that the metabolic activity of chondrocytes on different types of hydrogels gradually increased from day one to day five (Figure 5). Among all of the hydrogels, the P50/S50 hydrogel showed the highest cell growth over the culture period. These results indicated that the P50/S50 hydrogel provided an optimum environment for chondrocyte growth. However, cells on P75/S25 showed the lowest proliferation among the composite groups over the incubation period. The reason for this result might be that the higher hydrophilicity of PVA gels makes cellular adhesion difficult [56].

As the P50/S50 exhibited interconnected porous structure and higher level of cell growth compared to two other (P75/S25 and P25/S75) blended hydrogels, we selected the P50/S50 hydrogel for both in vitro and in vivo chondrogenesis studies. In our in vitro chondrogenesis study, the H&E and MT staining results indicated that the chondrocytes located within the newly formed cartilage matrix and immature chondrocyte lacunar structure (Figure 6) had morphological characteristics similar to those of native cartilage tissue [57,58]. It was also observed that the number of lacuna increased from four weeks to six weeks (Figure 6), indicating that cartilage maturity gradually increased over time. Graft rejection is a major concern associated with the transplantation of cells/tissue/scaffolds into host animals due to inadequate histocompatibility [59]. For in vivo cartilage engineering, isolated rat chondrocytes were seeded on the hydrogel and then implanted into the same rat. We observed an intact auricular cartilage without any graft rejection and immunogenic reactions six weeks after implantation. The ear-shaped hydrogels were also observed to retain their mechanical properties such as flexibility and elasticity (Figure 7). From this observation, it can be concluded that PVA/SF composite ear-shaped hydrogel provided sufficient mechanical strength to maintain its original 3D structure throughout the culture period. Biodegradability is another important factor, along with the mechanical strength, to control the 3D fate of engineered tissue [10]. A rapidly degrading scaffold does not provide sufficient structural support for new tissue formation. The degradation rate of an ideal scaffold should be balanced with neo-tissue formation [9,10]. Our histological finding suggested that the degradation rate of the hydrogel was balanced with neo-cartilage formation both in vitro and in vivo. The amount of residual hydrogel in the engineered cartilage decreased as neo-auricular cartilage formation and maturation increased throughout the experimental period (Figure 6 and Figure 8B,C). We also found no significant differences in cartilage formation and maturation level in the different regions of the engineered auricular cartilage at the same time point. These findings collectively indicated that auricular cartilage development and maturation is a time-dependent process.

## 4. Materials and Methods

### 4.1. Preparation of SF and PVA Solutions

*Bombyx mori* cocoons were degummed to remove the sericinesby boiling for 1 h in 0.02 M Na_2_CO_3_ solution. The degummed silk was washed in distilled water and then dried in an oven. Subsequently, the degummed silk fibers were dissolved in CaCl_2_, ethanol, and distilled water at a molar ratio of 1:2:8, respectively, at 95 mL for 1 h. Then, the prepared solution was filtered through Mira cloth (Calbiochem, San Diego, CA, USA), and ions were removed through dialysis tubing with a molecular weight cut off 12,000 to 14,000 Da (Spectra/Por^®^, Rancho Dominguez, CA, USA) in distilled water. The final concentration of the SF in the solution was 6 wt %, which was confirmed by weighing the residual solid after drying. This solution was concentrated up to 10% *w*/*v* using poly (ethylene glycol) (PEG) and stored at 4 °C. PVA 10% *w*/*v* solution was prepared by dissolving PVA (*M*_w_ 9000–10,000, 80% hydrolyzed, Sigma Aldrich, St. Louis, MO, USA) in distilled water in a flask with continuous stirring at 80 °C for 2 h. Subsequently, it was cooled to room temperature for further uses.

### 4.2. Preparation of the 3D Ear Mold

The 3D ear mold was prepared with a Fuse Deposition Modeling (FDM) 3D printer (3DISON, Seoul, Korea) using PLA filaments. The human ear 3D modeling file was converted to a slicing program for 3D printing. A 3D ear negative mold was also prepared using silicone (GT Products, Inc., 501 Industrial Blvd, Grapevine, TX, USA).

### 4.3. Fabrication of Hydrogel

**Figure 9:**
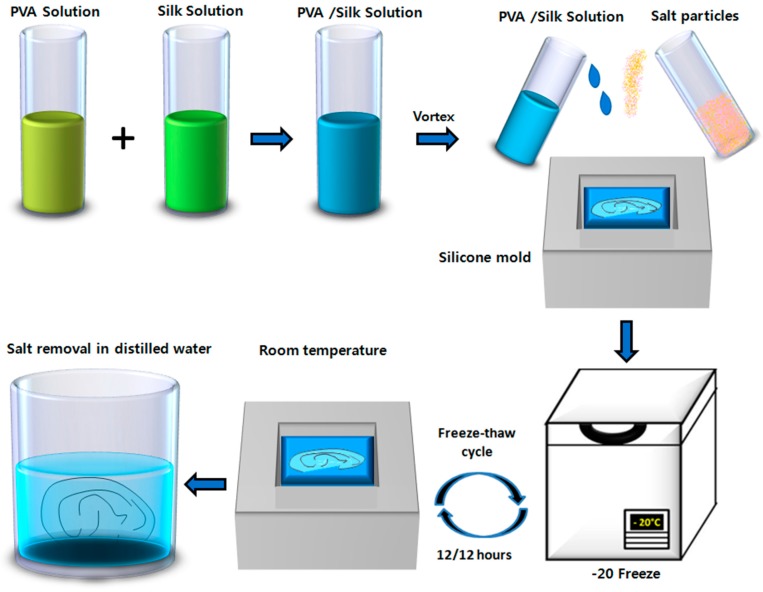
Schematic representation of the process for ear-shaped hydrogel fabrication. PVA and SF solutions were prepared by dissolving them in distilled water. The PVA/SF composite was prepared. The ear-shaped hydrogel was fabricated using the salt leaching method by filling a silicone mold. Fabrication was carried out by subjecting them to freeze-thaw cycles, and finally, the salt was removed by placing them in distilled water.

We constructed five types of hydrogel with different ratios of PVA and SF: 100% PVA (P100), 75% PVA and 25% SF (P75/S25), 50% PVA and 50% SF (P50/S50), 25% PVA and 75% SF (P25/S75), and 100% SF (S100). The fabrication was performed by the salt leaching method [55]. The ear-shaped hydrogel was fabricated by filling the negative mold with only the P50/S50 blend and salt particles (Figure 9). Finally, the fabrication of the hydrogels and ear-sheped hydrogel was completed by subjecting them to freeze-thaw cycles (12/12 h) for 3 days. The freeze-thaw cycles induce physical cross-linking within the polymers [60]. Thereafter both the hydrogel and ear-shaped hydrogel were transferred to distilled water to remove the salt for three days.

### 4.4. Analysis of Mechanical Properties (Tensile Strenth)

The tensile strenth of hydrogels was analyzed using a QM 100S (Qmesys Corp., Kyounggi, Korea) equipped with the across-head speed of 1 mm/min. The samples were punched from a parallel portion of 20 mm in length and 2.5 mm in width. At least three specimens were tested from each group, and the average values were calculated.

### 4.5. Swelling Ratio and TGA Analysis

The swelling ratio was calculated according to an previously reported method [46]. Each hydrogel was immersed in distilled water at room temperature for 24 h. After that, the excess water was removed, and the wet weight (Ws) of each hydrogel was measured. The hydrogels were then dried in an oven at 60 °C under vacuum overnight, and the dry weight (Wd) of the hydrogel were measured. Finally, the swelling ratio of each hydrogel was calculated using following formula.

(1) $$Swellingratio=\;\frac{\left(\mathrm{Ws}-\mathrm{Wd}\right)}{\mathrm{Wd}}$$

The TGA of the hydrogels were analyzed (TA Instruments, Newcastle, DE, USA) by ramping the samples at 10 °C/min, and heating was started from 30 to 700 °C. Heating was followed under a continuous nitrogen purge of 100 mL/min, and spectra were collected using Q600 Software (TA Instruments).

### 4.6. SEM and FTIR Analysis

The structure of the hydrogels were analyzed with a variable pressure field emission scanning electron microscope (VP-FE-SEM) (S-3500N, Hitachi, Tokyo, Japan). All samples were coated with a 10-nm layer of gold/palladium for 120 s with a discharge current of 15 mA using an Ion Sputter 1010 (Hitachi, Tokyo, Japan). The average pore sizes were measured after determining the average size of random pores (50 individual pores per sample) from SEM images (Figure 3C) using INNERVIEW 2.0 software. FTIR analysis was conducted using a BIO-RAD Excalibur Series FTIR Spectrometer (Cambridge, MA, USA). The spectra were recorded from 2000 to 600 cm^−1^ with a 2 cm^−1^ resolution and 32 scans.

### 4.7. Chondrocyte Isolation and Culture 

Auricular cartilage was harvested from the ear of a male Sprague-Dawley rat under aseptic conditions. Then, the cartilage sheet was washed with phosphate buffered saline (PBS) and chopped into approximately 2-mm pieces. Pieces of the cartilage sheet were digested in Dulbecco's Modified Eagle’s Medium (DMEM) with collagenase for overnight at 37 °C. The residual cartilage sheet was removed from the petri dish. Adhered chondrocytes were then trypsinized producing cell suspension and the cells were washed with PBS. Then, cell suspension was filtered through a cell strainer (70-μm pore size, SPL Life Sciences, Pocheon-si, Korea) and centrifuged for 5 min at 1000 rpm. Finally, the pellet was suspended and cultured in DMEM supplemented with 10% FBS (fetal bovine serum) and 1% A/A (antibiotics/antimycotics).

### 4.8. Cell Seeded Hydrogels

Each hydrogel (6 mm size) was sterilized in 70% ethanol. Then, the hydrogels were seeded with 2 × 10^4^ chondrocytes per hydrogel with 50 μL medium in 96-well culture plates. Subsequently, after an hour for attachment, 150 μL fresh medium was added to each well followed by incubation at 37 °C with 5% CO_2_. The cytotoxicity of the hydrogel was measured using a CCK-8 assay kit (Enzo Life Sciences, Farmingdale, NY, USA) after one, three, and five days of culture. Absorbance was measured at 450 nm by a microplate reader (Molecular Devices, Sunnyvale, CA, USA). For in vitro chondrogenesis, cell-seeded P50/S50 hydrogels were cultured for 2, 4, and 6 weeks. Then, the harvested hydrogels were fixed in 4% paraformaldehyde. The hydrogels were embedded with Optimum Cutting Temperature (OCT) (Leica Biosystems Melbourne Pty Ltd., Melbourne, Australia) compound and frozen at −80 °C. The specimens were sectioned at a 5-μm thickness and stained with Hematoxylin and eosin (H&E) and Masson’s Trichrome (MT).

### 4.9. Animal Study

Animal studies were carried out following the guidelines and with the approval of the Institutional Animal Care and Use Committee of Hallym University. The adult male Sprague-Dawley rats weighing 250–270 g each was housed in separate plastic cages with free access to laboratory rodent food and water under appropriate air and light conditions. The ear-shaped hydrogels were seeded with 2 mL of cell suspension at a density of 1 × 10^7^ cells/mL and were incubated for one day. Then, the hydrogels were implanted subcutaneously into rats. After 2, 4, and 6 weeks, the ratswere sacrificed, and implants were harvested. The harvested ear-shaped hydrogels were fixed in 4% paraformaldehyde. The hydrogels were divided into three pieces (Figure 8A) and embedded in optimum cutting temperature (OCT) compound followed by freezing at −80 °C. The specimens were sectioned at a 5-μm thickness and were stained with H&E and MT.

### 4.10. Statistical Analysis

Data are shown as the mean ± SEM from at least three independent experiments and were analyzed using GraphPad Prism (GraphPad software, San Diego, CA, USA) followed by one-way analysis of variance with Dunnett’s post-hoc test. Significant differences are presented as * *p* < 0.05, and ns indicates not significant.

## 5. Conclusions

This study demonstrates a precise method for engineering an ear-shaped cartilage using a PVA/SF hydrogel. The P50/S50 hydrogel showed reasonable porosity and water-binding abilities to provide a suitable environment for chondrocyte growth and the regeneration of auricular cartilage both in vitro and in vivo. This hydrogel also offered sufficient mechanical strength during the entire period of its engineering and did not induce graft rejection or immunogenic reactions. Histological observations revealed that mature cartilage with a typical lacunar structure formed within six weeks of culture implants. In future studies, we will investigate the deposition rate of different glycoproteins in neocartilage at various times and maturity levels. Thus, the process developed in this study could be a promising method for auricular cartilage engineering and external ear reconstruction.
